# Establishment and characterization of a sigmoid colon cancer organoid with spinal metastasis

**DOI:** 10.3389/fcell.2024.1510264

**Published:** 2025-01-03

**Authors:** Jingyao Chen, Shumin Cheng, Liang Gu, Zhangsen Huang, Changhua Zhang, Chunhui Sun, Songyao Chen

**Affiliations:** ^1^ Digestive Diseases Center, The Seventh Affiliated Hospital of Sun Yat-sen University, Shenzhen, China; ^2^ Guangdong Provincial Key Laboratory of Digestive Cancer Research, The Seventh Affiliated Hospital of Sun Yat-sen University, Shenzhen, China; ^3^ Scientific Research Center, The Seventh Affiliated Hospital of Sun Yat-sen University, Shenzhen, China

**Keywords:** sigmoid colon cancer, spinal metastases, genomic features, organoid culture, drug screening

## Abstract

**Background:**

Sigmoid colon cancer with spinal metastases is rare in distant metastasis. In addition, the prognosis of colon cancer patients with spinal metastases is extremely poor. In order to find effective therapeutic agents, we need to know the biological characteristics of such patients from related models.

**Methods:**

We collected sigmoid colon cancer tissue from a young female subject who was diagnosed with sigmoid colon cancer with multiple spinal metastases. We successfully established a sigmoid colon cancer organoid using this tissue and investigated drug screening in the patient. HE staining, immunohistochemistry, and DNA sequencing were utilized to compare the biological characteristics between the original tumor and the organoid. Furthermore, we investigated the drug screening of the sigmoid colon cancer organoid *in vitro*.

**Results:**

A colon cancer organoid from sigmoid colon cancer with spinal metastases was successfully established. The organoid culture maintained the morphological features, histological features, and genomic landscape of the corresponding sigmoid colon cancer cells. Moreover, we performed drug screening tests to evaluate the effects of chemotherapeutic drugs and targeted drugs.

**Conclusion:**

The sigmoid colon cancer organoid with spinal metastases was a favorable preclinical model to explore the clinicopathologic characteristics of colon cancer patients with spinal metastases.

## Introduction

Osseous metastasis originating from colorectal cancer (CRC) is extremely rare. It is reported that only 1.1%–6.12% of CRC cancer patients have bone metastasis (Baek et al., 2016; Lei et al., 2019). Patients with osseous metastases originating from CRC have a poor prognosis. In addition, many skeletal-related events (SREs) such as severe bone pain, pathologic fracture, spinal compression fracture, and hypercalcemia may occur in cases of osseous metastasis (Ganry et al., 2008). These SREs severely impair the patient’s quality of life. A single lesion of bone metastasis can be treated by surgery, ablation, or radiation. However, it is hard to cure multiple bone metastases with surgery and radiation (Gdowski et al., 2017).

Once osseous metastasis has taken place, it is hard to cure the disseminated tumor cells. Management of CRC patients with osseous metastasis needs a multidisciplinary team approach to the treatment of the primary tumor with anti-cancer therapy, including surgery, chemotherapy, radiation therapy, and immunotherapy (Gdowski et al., 2017). Despite such treatments for osseous metastases, patients have an awful prognosis. According to literature review, the median survival time of CRC patients with bone metastasis is approximately 5 months (Kawamura et al., 2018; Lei et al., 2019). Therefore, an urgent and effective treatment method needs to be researched for this kind of terminal-stage cancer.

Animal experiments can be carried out in order to find the correct drugs. However, a series of ethical issues and long culture periods hinder the development of research. An emerging three-dimensional (3D) *in vitro* culture system called “patient-derived tumor organoid (PDO)” has been devoted to recapitulating the characteristics of anticancer drugs. Using a 3D patient-derived organoid culture system and creating tumor organoid biobanks will definitely expand the types of patient samples that can be researched (Tuveson and Clevers, 2019). From overviewing the published literature reports, PDO culture has been established stably from different kinds of cancers, including colorectal cancer (van de Wetering et al., 2015), gastric cancer (Nanki et al., 2018), prostate cancer (Gao et al., 2014), renal cancer (Calandrini et al., 2020), breast cancer (Sachs et al., 2018), hepatocellular carcinoma and cholangiocarcinoma (Broutier et al., 2017), pancreatic cancer (Driehuis et al., 2019), bladder cancer (Lee et al., 2018), ovarian cancer (Kopper et al., 2019), and lung cancer (Sachs et al., 2019). The broad application of PDO is drug screening, which mostly mimics the response of these patients to the same drugs (Pauli et al., 2017). More importantly, drug screening results from PDO can be used for personalized medicine programs (Vlachogiannis et al., 2018).

Here, we report a case of bone metastasis originating from sigmoid colon cancer in a young woman, in which we successfully established cultures of the primary sigmoid carcinoma-derived organoid. Then, we showed that the sigmoid colon carcinoma-derived organoid recaptured the features of cancer cells in the original tumor tissue, including morphology, histology, gene mutation, and expression profile. In addition, we explored the potential of using organoids as a preclinical drug screening model to optimize chemotherapy in the treatment of sigmoid colon cancer with bone metastasis.

## Materials and methods

### Patient and sample collection

Primary sigmoid colon cancer tissue was collected from a 29-year-old female Chinese patient during palliative surgery. The sigmoid colon cancer tissue was cut into small pieces and washed three times with cold phosphate-buffered saline (PBS) with penicillin/streptomycin (Thermo Fisher Scientific, cat#15140122). Then, 1 mL digestion buffer (Biosharp, cat#BL501A), 100 µL dispase II buffer (Macklin, cat#D923682), 10 µL DNaseI (Beyotime, cat#D7073), and 1 µL Y27632 (MCE, cat# HY-10071) were added to a 1.5-mL centrifuge tube. The tube was placed in a water bath at 37°C for 30 min. After that, isolated tumor cells from the above buffer were then embedded in Matrigel (Corning, cat#356237) on ice and seeded into 24-well plates. The Matrigel was polymerized for 15 min at 37°C, and 500 µL/well of culture medium (Supplementary Table S1) was overlaid. The plates of cells were then incubated at 37°C and 5% CO_2_. The culture medium was refreshed every 2 days.

Two other small pieces of tumor tissue were quickly frozen with liquid nitrogen for DNA sequencing. The remainder was fixed in formalin for immunohistochemistry. Sigmoid colon cancer tissue was obtained with informed consent. This study was approved by the Ethical Committee of the Seventh Affiliated Hospital of Sun Yat-sen University (No. KY-2020-024-02).

### Histology and immunohistochemistry

We cultured the organoid for 10 days after passage. Then, the organoid with Matrigel was washed with PBS, fixed in 4% paraformaldehyde, and then embedded in paraffin. Hematoxylin and eosin (H&E) staining was performed as described previously. Immunohistochemical staining was used to evaluate the protein expression. The slides were incubated with anti-MLH1 (1:100, Abcam, cat# ab223844), anti-MSH2 (1:8000, Abcam, cat# ab227941), anti-MSH6 (1:500, Abcam, cat# ab92471), anti-PMS2 (1:600, Abcam, cat# ab110638), anti-Ki-67 (1:500, Abcam, cat# ab243878), and anti-HER2 (1:1600, Abcam, cat# ab134182) antibodies.

### DNA isolation and DNA sequencing

We cultured the organoid for 10 days after passage. Then, we harvested the organoid for DNA extraction. Genomic DNA was extracted by using a QIAamp DNA mini kit (QIAGEN, 51304), according to the set instructions. Exome sequencing was also performed on the matched peripheral blood mononuclear cells for comparison. DNA quality was controlled by a high-sensitivity DNA assay. Based on next-generation sequencing, the DNA libraries were sequenced on the Illumina HiSeq platform, and 150 bp paired-end reads were generated. The reads were filtered by the setting rules to gain high-quality clean reads by fastp (Chen et al., 2018). Then, the paired-end clean reads were mapped to the human reference genome (GRCh37/hg19) using Burrows-Wheeler Aligner (BWA) (Li and Durbin, 2010).

### Mutational signature analysis

We employed the SigProfiler suite to perform a comprehensive mutational signature analysis. The analysis was conducted in three main steps:

SigProfilerExtractor: we first extracted *de novo* mutational signatures from the mutation data formatted as variant call format (VCF). This tool identified unique mutational patterns within our samples, including single-base substitutions (SBS), doublet-base substitutions (DBS), and small insertions/deletions (indels).

SigProfilerMatrixGenerator: next, we utilized SigProfilerMatrixGenerator to create a mutation matrix from the extracted mutation data. This matrix served as a structured representation of the mutations, allowing for further comparative analysis.

SigProfilerAssignment: finally, we matched and compared the extracted mutational signatures with those available in the Catalog of Somatic Mutations in Cancer (COSMIC) database. By calculating the cosine similarity between our *de novo* signatures and existing COSMIC signatures, we identified known mutational processes relevant to our samples.

### Somatic mutation analysis

We used GATK Mutect2 (Broad Institute) (DePristo et al., 2011) to identify single nucleotide variants (SNVs) and small insertions/deletions. The criteria for positive events were as follows: passing the quality check, median base not less than 20, median mapping quality not less than 30, supporting reads not less than four, and allele frequency less than 0.1%. Moreover, the exclusive criteria were variants that appeared in a large proportion of genes and in highly paralogous genes.

### Identification of driver genes

We used the R package “dNdScv” (Martincorena et al., 2017) to identify cancer driver genes. The dNdScv algorithm works by comparing the observed and expected rates of non-synonymous and synonymous mutations, accounting for differences in mutation context and gene length. We used VCF files generated by Mutect2, with GRCh37 as the reference genome. To control for multiple testing, the Benjamini–Hochberg correction was applied, and mutations with a q-value less than 0.1 were regarded as significant.

### Germline mutation analysis and somatic copy number variant analysis

Germline mutation analysis and somatic copy number variants (CNVs) were confirmed by using FACETS (Arora et al., 2022) from the standardized data on WES. GISTIC2.0 was used to visualize the copy number amplifications and deletions of each specimen.

### Pathway enrichment analysis

Metascape (http://metascape.org/) was used to perform pathway enrichment analysis. We collected the genes of CNVs and subsequently performed multiple pathway enrichment analyses through Metascape, including GO, KEGG, Reactome gene set, CORUM, and WikiPathways. The top 20 pathways are shown in this study.

### Cell culture

In this study, we purchased human colon cancer cells SW480 and LoVo from the American Type Culture Collection (ATCC). SW480 and LoVo cells were cultured in DMEM medium supplemented with 10% fetal bovine serum (FBS) and 1% penicillin and streptomycin solution in a cell incubator at 37°C and 5% CO_2_.

### Drug screening

After the sigmoid colon cancer-devoid organoid’s stable growth and passage, we harvested it and dissociated it into single cells. Then, we calculated the total number of cells by using a cell counting plate. A total of 3,000 cells per well were seeded into 96-well cell culture plates (Corning) by the on-top method. The 96-well plates were incubated for 24 h at 37°C and 5% CO_2_ in a cell culture incubator. After that, media was removed and replaced with 100 µL of drug-containing complete colon cancer organoid medium. A total of 72 h after different drug treatments, the cell proliferation viability of the organoid was measured by cell counting kit-8, following the manufacturer’s instructions. Six different concentrations of each drug (chemotherapeutic drugs and targeted drugs) and three concentrations of monoclonal antibody drugs in the sigmoid colon cancer organoid were used to develop dose–response curves. Data on drug screening and 50% maximal inhibitory concentration (IC_50_) for each drug were analyzed using GraphPad Prism 8.

## Results

### Case report

**FIGURE 1:**
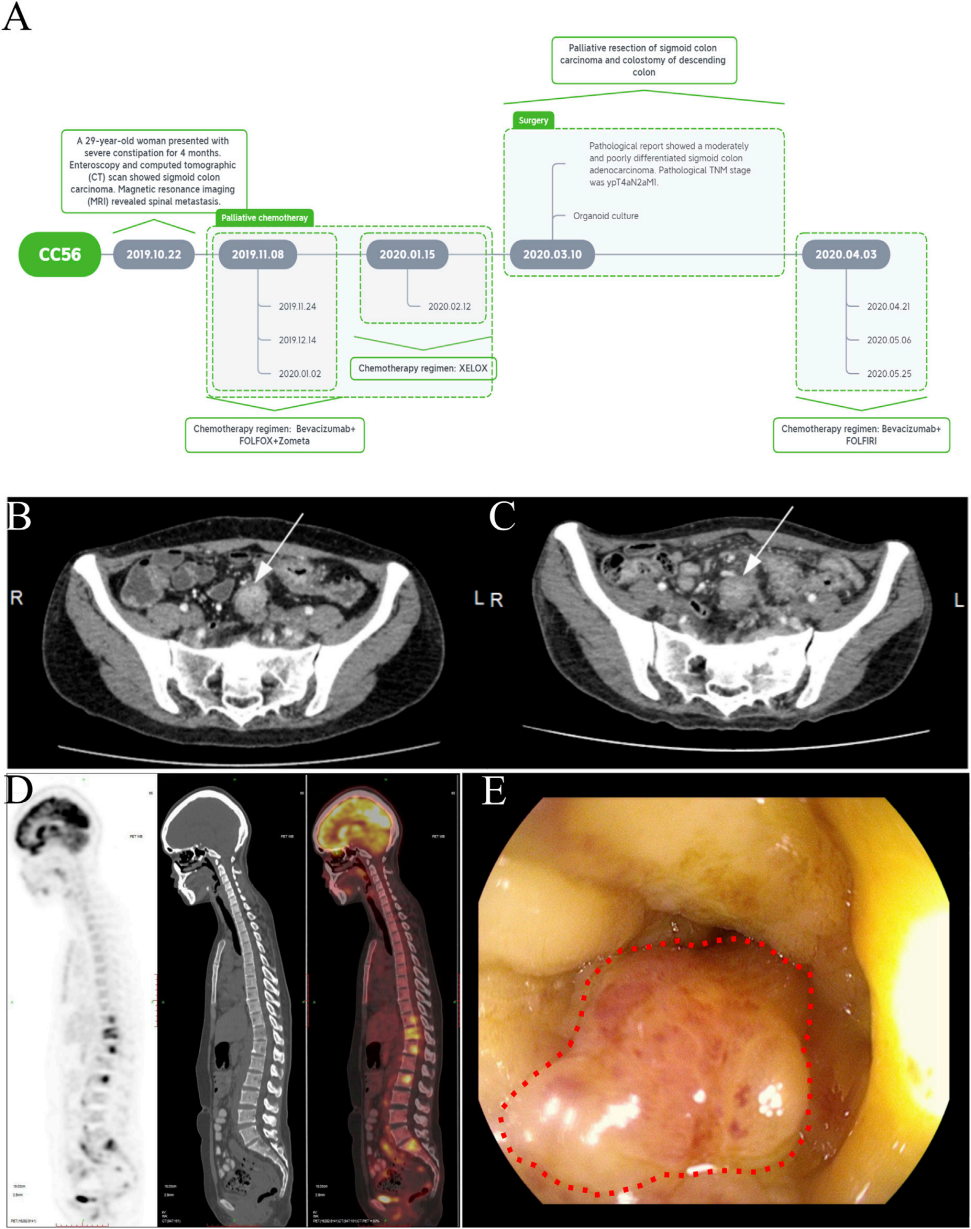
Treatment timeline and clinical characteristics of a case of primary advanced sigmoid colon cancer with bone metastasis. **(A)** Treatment timeline B-C. Abdominal computed tomography showing sigmoid colon carcinoma before **(B)** and after treatment **(C)** (white arrow). **(D)** PET-CT showing the multiple spinal metastases. **(E)** Enteroscopic image of stenosis caused by sigmoid colon carcinoma (red dotted circle).

A 29-year-old woman presented with a 4-month history of constipation. The treatment timeline is shown in Figure 1A. A multidisciplinary team recommended chemotherapy. The patient received four cycles of FOLFOX (5-fluorouracil, leucovorin, and oxaliplatin) combined with bevacizumab and zoledronic starting on 8 November 2019. Due to the patient’s adverse effect of severe vomiting, she started to receive two cycles of the XELOX regimen (capecitabine and oxaliplatin) starting on 15 January 2020. After that, the patient was readmitted for evaluation of the response to chemotherapy on 7 March 2020. Unfortunately, the enhanced CT and MRI showed no significant change in tumor after palliative chemotherapy (Figures 1B, C). The positron emission tomography/computed tomography (PET/CT) scan showed sigmoid colon cancer with multiple lymph node metastases and thoracic, lumbar, iliac, and pubic bone metastases (Figure 1D). An endoscopic examination that was performed to examine the rectum and colon showed luminal stenosis of the sigmoid colon so that the examination of endoscopy was interrupted (Figure 1E). Pathological examination of mucosal biopsies from the lesions revealed adenocarcinoma. Immunohistochemical (IHC) staining was performed and demonstrated CK(+), CEA (+), CK7(−), CK20(+), CDX-2 (+), and SATB-2 (+). Subsequently, palliative tumor resection and colostomy of the descending colon were proposed and completed. Pathological examination showed a moderately–poorly differentiated adenocarcinoma (ypT4aN2aM1). IHC revealed HER2(1+), MLH1 (+), PMS2(+), MSH2(+), MSH6(+), and Ki67 expression in approximately 65% of the tumor cells. The patient’s recovery was not very smooth because of wound complications and spinal pain. Therefore, she started to accept the chemotherapy regimen of FOLFIRI (5-fluorouracil, leucovorin, and irinotecan) combined with bevacizumab on 3 April 2020. Subsequently, the patient accepted three cycles of FOLFIRI combined with the bevacizumab regimen on 21 April, 6 May, and 25 May, respectively (Figure 1A). However, the chemotherapy did not relieve the bone pain, and disease progression occurred. On 16 June, the CT scan showed larger and wider lesions of spinal metastases than before.

### Establishment of the sigmoid colon carcinoma organoid

**FIGURE 2:**
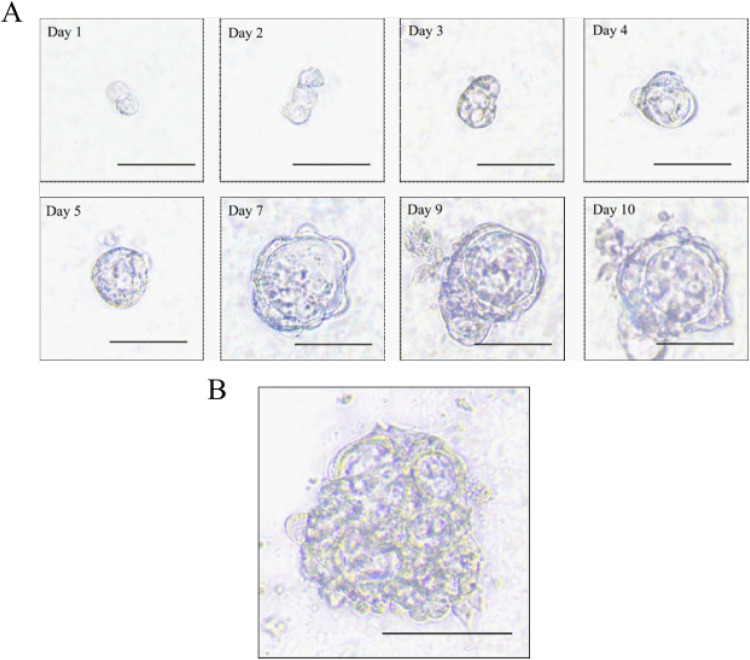
Morphology of the sigmoid colon carcinoma organoid. **(A)** Time course of the sigmoid colon carcinoma organoid culture (scale bar, 50 μm). **(B)** Multiple lumen structure of the sigmoid colon carcinoma organoid (scale bar, 50 μm).

We obtained sigmoid colon cancer tissue from the patient who underwent palliative sigmoid colon resection. We washed the sample with PBS and removed normal mucosa. Then, the tumor tissue was cut into small pieces so that we could isolate tumor cells through digestion. We obtained isolated cells and resuspended them with Matrigel. Finally, we seeded them in 24-well plates with the organoid culture medium. We recorded the time course of the sigmoid colon cancer organoid culture (Figure 2A). Interestingly, we observed the multiple lumen structure of the organoid during the culture period (Figure 2B).

### Histopathologic and molecular characteristics are retained in sigmoid colon cancer organoid

**FIGURE 3:**
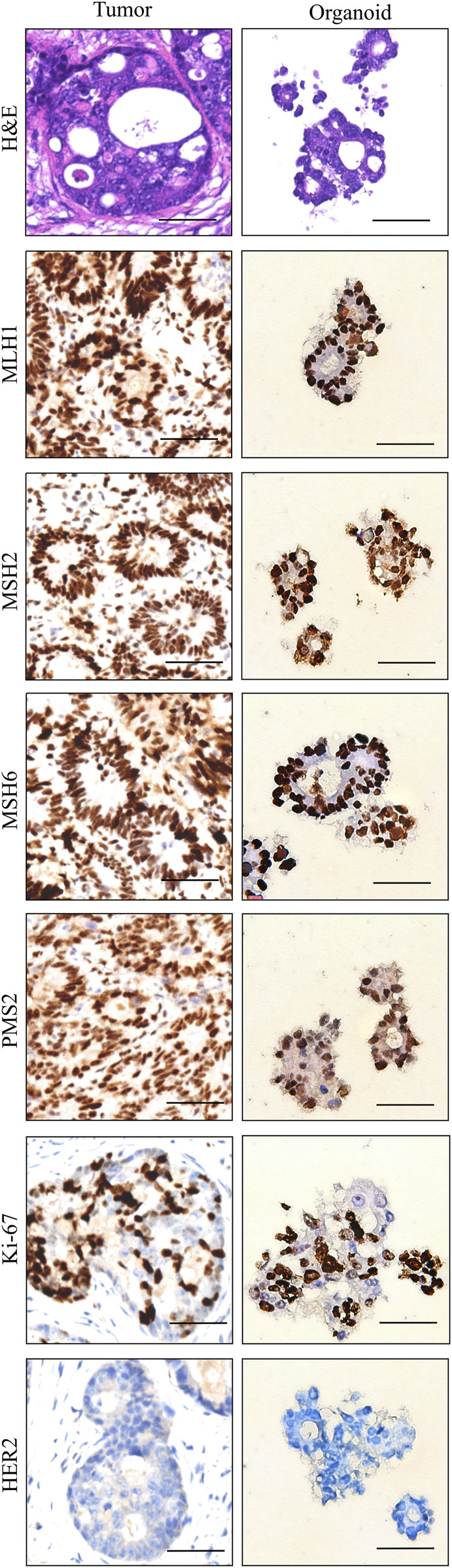
Histopathology and characterization of sigmoid colon carcinoma tissue (left column) and the organoid (right column). Scale bar, 50 μm.

We examined the paraffin-embedded tumor tissue and the patient-derived sigmoid colon cancer organoid morphologically. Hematoxylin and eosin staining demonstrated that the sigmoid colon cancer organoid preserved the histopathological features of the original tumor tissue. They showed a similar structure of specific glandular features and a similar degree of differentiation. For further confirmation of histological characteristic preservation, several important biomarkers need to be examined. Microsatellite instability (MSI) markers such as MLH1, MSH2, MSH6, and PMS2 can reflect the prognosis of colorectal patients. The patient can be defined as having microsatellite instability-high status by IHC staining of tumor tissue. The above four MSI markers were also detected in the organoid, which was consistent with the status of the tumor tissue (Figure 3). Ki67 is a cell proliferation marker. Ki67 was detected in approximately 65% of the cells in the tumor tissue and the organoid. HER2 is a member of the EGFR family of proteins. Amplification or mutation of ERBB2 plays an important role in targeted treatment. From the IHC result of both tumor tissue and organoid, HER2 expression was weak. In summary, we conclude that organoids recapitulate the biological features of the primary tissue (Figure 3).

### Mutation signature

**FIGURE 4:**
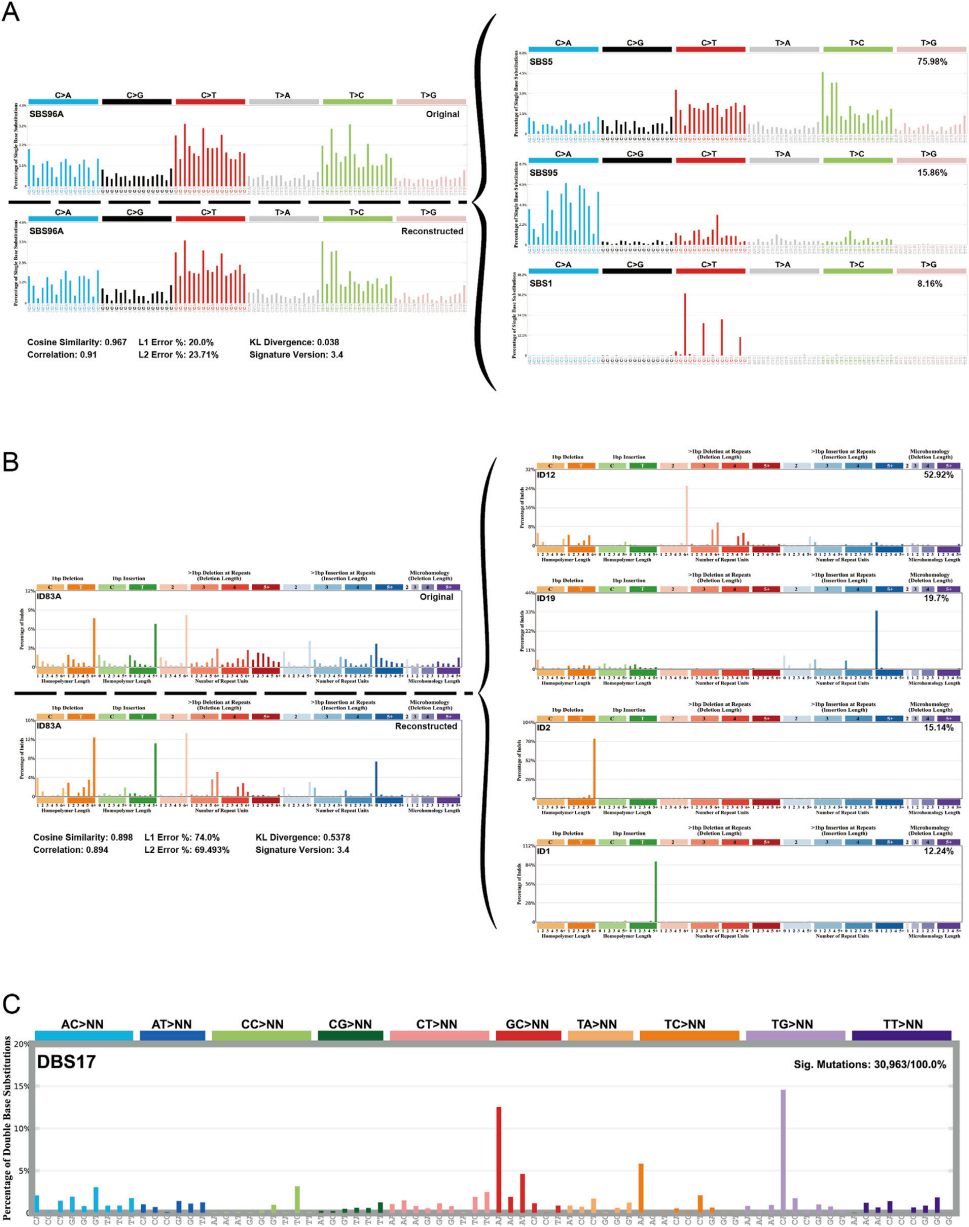
Mutational signature of the sigmoid colon carcinoma tissue, organoid, and blood sample. **(A)** *De novo* signature SBS96A was identified in three samples and highly correlated with combinations of existing COSMIC signatures. **(B)** ID feature ID83A was identified in three samples and was highly correlated with combinations of existing COSMIC features. **(C)** A new *de novo* DBS feature, called feature DBS17, was found that was not mapped to any COSMIC DBS feature.

In order to identify the mutation signature of sigmoid colon cancer tissue, organoid, and blood sample, we used SigProfiler to identify the *de novo* signature, an indel signature that was compared with COSMIC. As a result, the *de novo* signature SBS96A was identified and highly correlated with combinations of existing COSMIC signatures (cosine similarity: 0.967). SBS5 (75.98%), SBS95 (15.86%), and SBS1 (8.16%) contributed to this *de novo* SBS signature (Figure 4A). The ID signature ID83A was also identified and highly correlated with the comparison of existing COSMIC signatures (cosine similarity: 0.898). Among them, ID12 (52.92%), ID19 (19.7%), ID2 (15.14%), and ID1 (12.24%) contributed to this ID signature (Figure 4B). In addition, we found a novel *de novo* signature of DBS, DBS17, which is not mapped to any of the COSMIC DBS signatures (Figure 4C).

SBS1 and SBS5 are often identified in multiple cancer types, especially in tumors with increased activity of APOBEC family enzymes (Nunes et al., 2024). The high proportion of this signature may reflect the presence of APOBEC-induced mutations in our samples, suggesting possible therapeutic targets or prognostic indicators. SBS95 is associated with specific environmental factors and the decreased ability to repair DNA damage, and it is usually more prominent in skin cancer (Degasperi et al., 2022). This suggests that our samples may be related to environmental factors or patient lifestyle.

The newly discovered DBS signature DBS17 does not correspond to any known signature in the COSMIC database, and it may represent a distinct mutational process that has not been previously characterized. This uniqueness may imply a novel mechanism underlying mutational events in our samples, possibly driven by specific environmental exposures, metabolic processes, or tissue-specific factors.

### Features of somatic variant and driver genes

**FIGURE 5:**
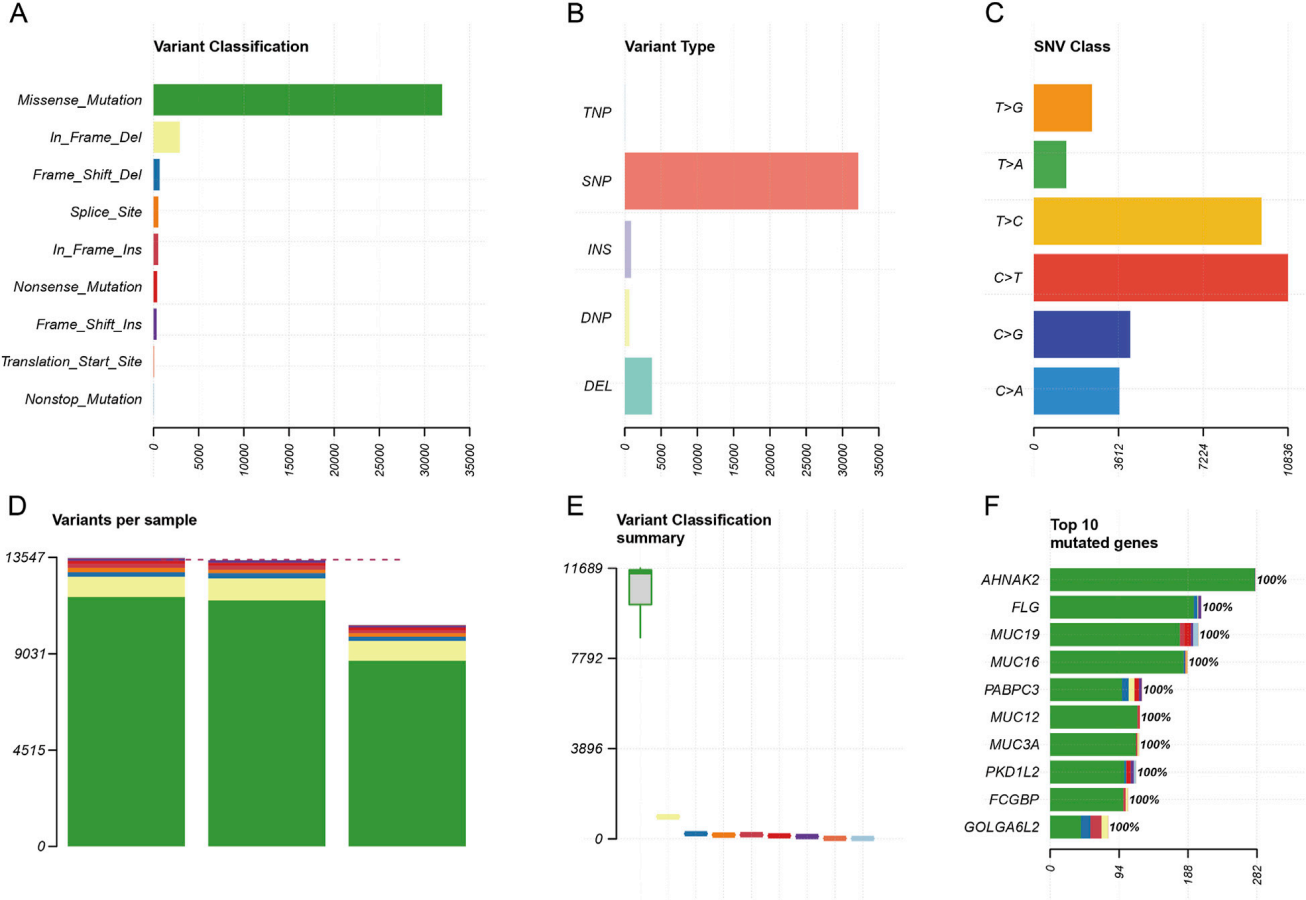
Somatic variant feature analysis. **(A)** Variant classification analysis. **(B)** Variant type analysis. **(C)** Class of point mutation analysis. **(D)** Number of mutations per sample analysis (left to right: blood, tissue, and organoid). **(E)** Variant classification summary. **(F)** Top 10 mutated genes.

We analyzed the characteristics of the variation from aspects that included, among others, categorization, type, model of point mutation, and quantity of mutations. From these three specimens, we discovered that missense mutations and in-frame deletions were the majority of categorization types (Figure 5A). SNPs were the main variant type (Figure 5B). The frequency type of SNV classes from high to low is C > T, T > C, C > G, and so on (Figure 5C). The number of mutations per sample from high to low was in the blood, tissue, and organoid (Figure 5D). The variant classification summary is shown in Figure 5E. The top 10 mutated genes are also shown in Figure 5F: *AHNAK2*, *FLG*, *MUC19*, *MUC16*, *PABPC3*, *MUC12*, *MUC3A*, *PDK1L2*, *FCGBP*, and *GOLGA6L2*.

Driver gene analysis also demonstrated that *SLC9B1*, *PDZRN3*, *PABPC3*, *ASPM*, *ZNF419*, *CMYA5*, *CALHM4*, *KMT2C*, *MS4A14*, *USP17L22*, *DIP2C*, *WDPCP*, *GRM7*, *NLRP12*, and *PGP* were the significant mutated genes in the blood, tissue, and organoid samples (Supplementary Figure S1).

### Germline mutation and somatic copy number variation

**FIGURE 6:**
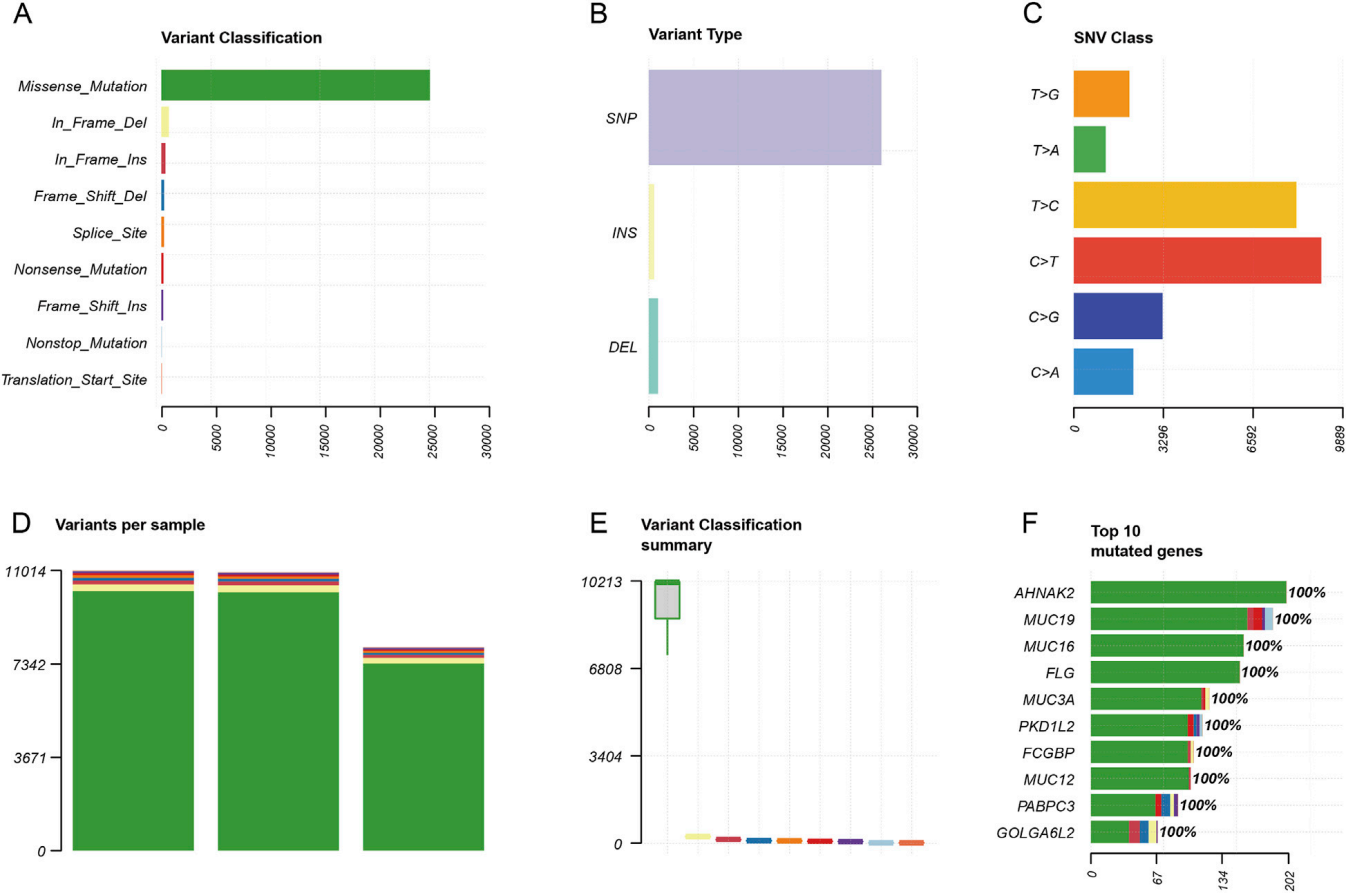
Germline mutation feature analysis. **(A)** Variant classification analysis. **(B)** Variant type analysis. **(C)** Class of point mutation analysis. **(D)** Number of mutations per sample analysis (left to right: blood, tissue, and organoid). **(E)** Variant classification summary. **(F)** Top 10 mutated genes.

Similarly, we also analyzed the germline mutation of categorization, type, model of the point mutation, and quantity. The result showed that the top of the germline mutation categorized was a missense mutation (Figure 6A). The main variant type was SNP (Figure 6B). The top three SNV classes were C > T, T > C, and C > G (Figure 6C). The number of variants per sample from high to low was in the blood, tissue, and organoid (Figure 6D). The variant classification summary is shown in Figure 6E. The top 10 mutated genes are *AHNAK2*, *MUC19*, *MUC16*, *FLG*, *MUC3A*, *PKD1L2*, *FCGBP*, *MUC12*, *PABPC3*, and *GOLGA6L2* (Figure 6F).

**FIGURE 7:**
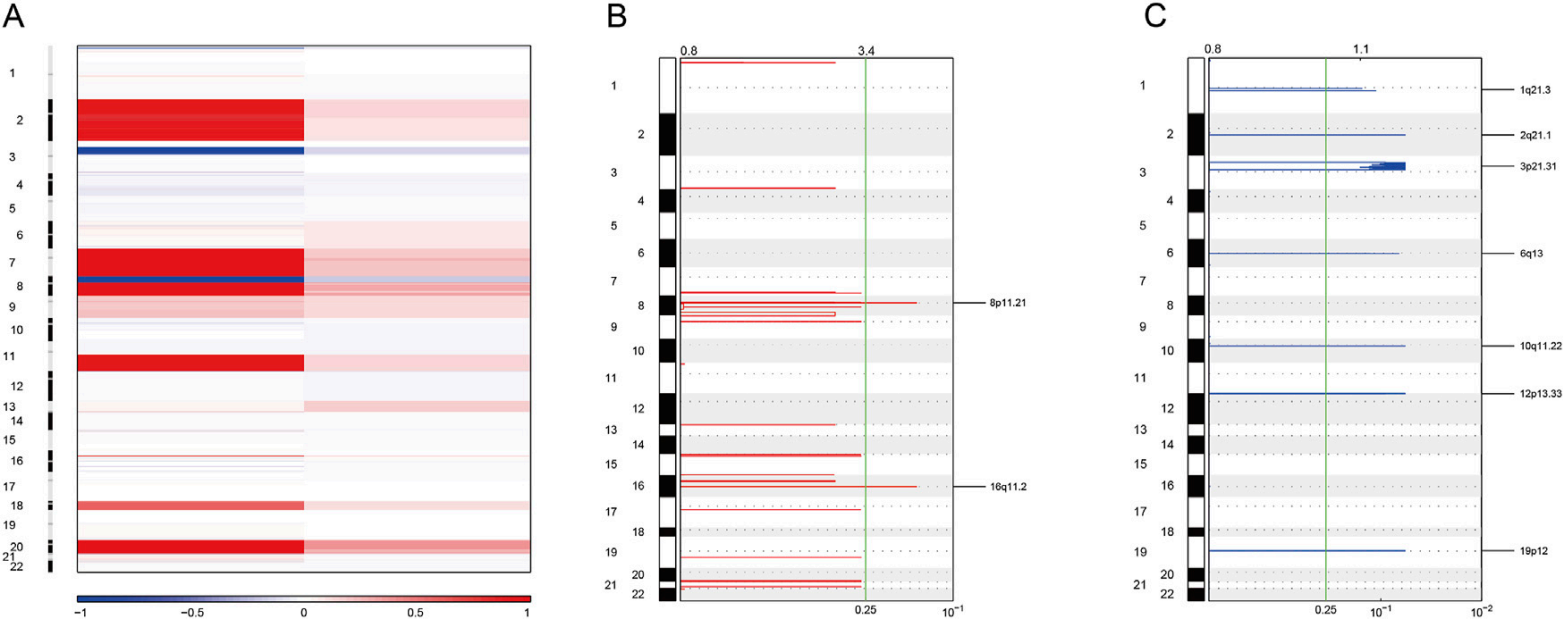
Somatic copy number variation analysis. **(A)** Heatmaps showing copy number amplifications (red) and deletions (blue) of standardized data for WES. **(B)** GISTIC2 figure showing significant local amplifications, including 8p11.21 and 16q11.2. **(C)** GISTIC2 map showing significant local deletions, including 1q21.3 and 2q21.1.

As for copy number variation analysis, we used blood samples for control. The heatmap was applied to identify the copy number amplification and deletion (Figure 7A). As a result, we found that the copy number was observably amplified in 8p11.21 and 16q11.2 (Figure 7B). The result also showed that the copy number was observably deleted in 1q21.3 and 2q21.1 (Figure 7C).

### Pathway enrichment analysis

**FIGURE 8:**
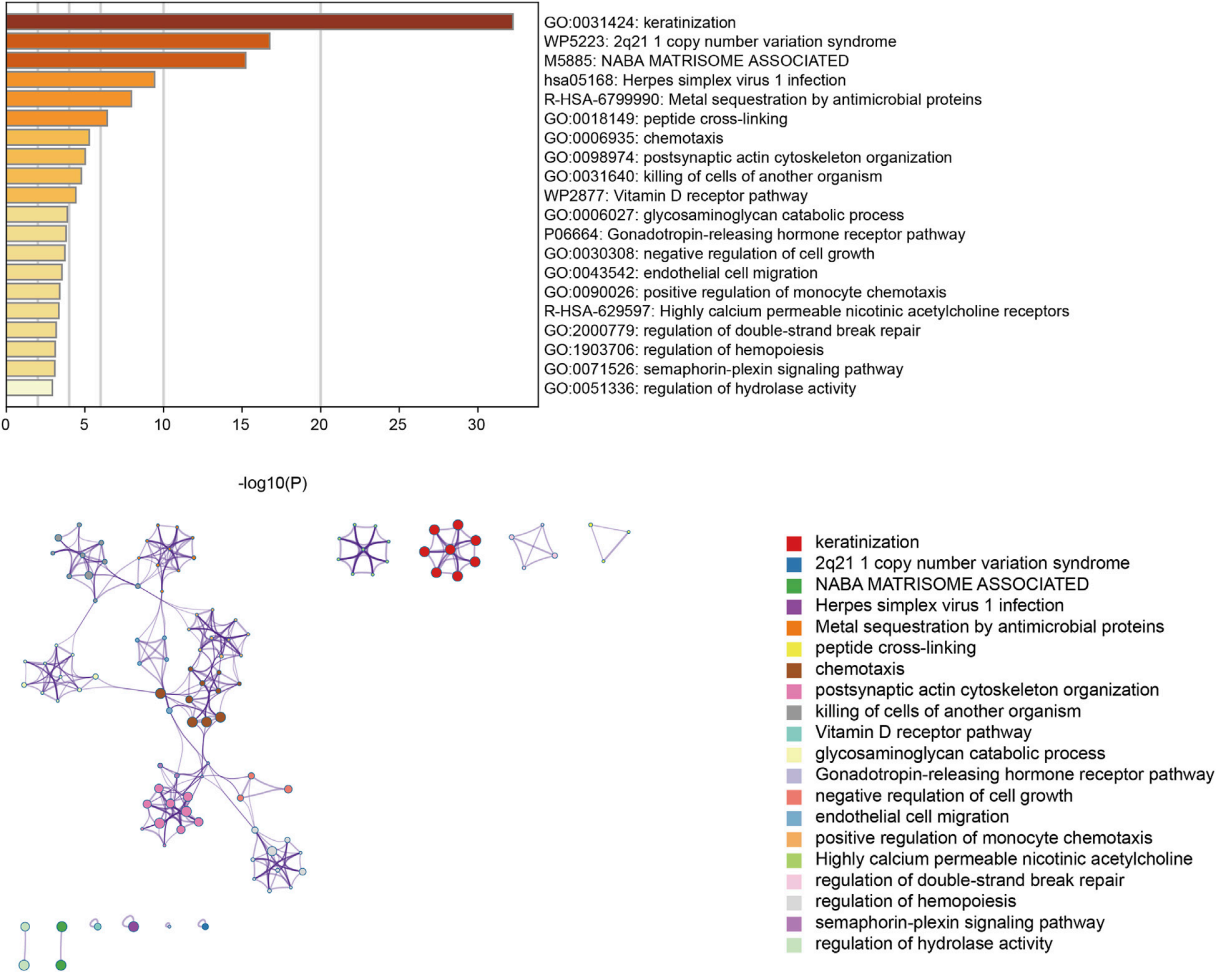
Enrichment pathway analysis utilizing the Metascape database.

Next, the CNV-related genes were used to perform pathway enrichment analysis in the Metascape database. The result showed enrichment pathways including keratinization, 2q21.1 copy number variation syndrome, NABA matrisome-associated herpes simplex virus infection, metal sequestration by antimicrobial proteins, peptide cross-linking, chemotaxis, postsynaptic actin cytoskeleton organization, killing of cells of another organism, and vitamin D receptor pathway (Figure 8).

### Drug screening

In order to evaluate the preclinical model, we tested different clinical drugs for colon cancer according to the NCCN guidelines including 5-fluorouracil, oxaliplatin, docetaxel, paclitaxel, cisplatin, epirubicin, and SN38. Several targeted drugs (including apatinib, lapatinib, alpelisib, regorafenib, and everolimus) and monoclonal antibodies such as cetuximab and trastuzumab were also chosen to be examined. Zoledronic acid, a drug used to treat osteolytic bone metastases, was used to treat the sigmoid colon cancer organoid. We aimed to generate dose–response inhibition curves to identify half-maximal inhibitory concentrations (IC_50_) by six concentrations of each drug. Organoid cultures were digested and plated on 96-well plates by the on-top method. After growing for 24 h, organoid cultures were treated with different concentration gradients of the above drugs. Then, after culturing for 72 h, organoid cultures were examined for cell viability by a Cell Counting Kit-8 (CCK-8).

**FIGURE 9:**
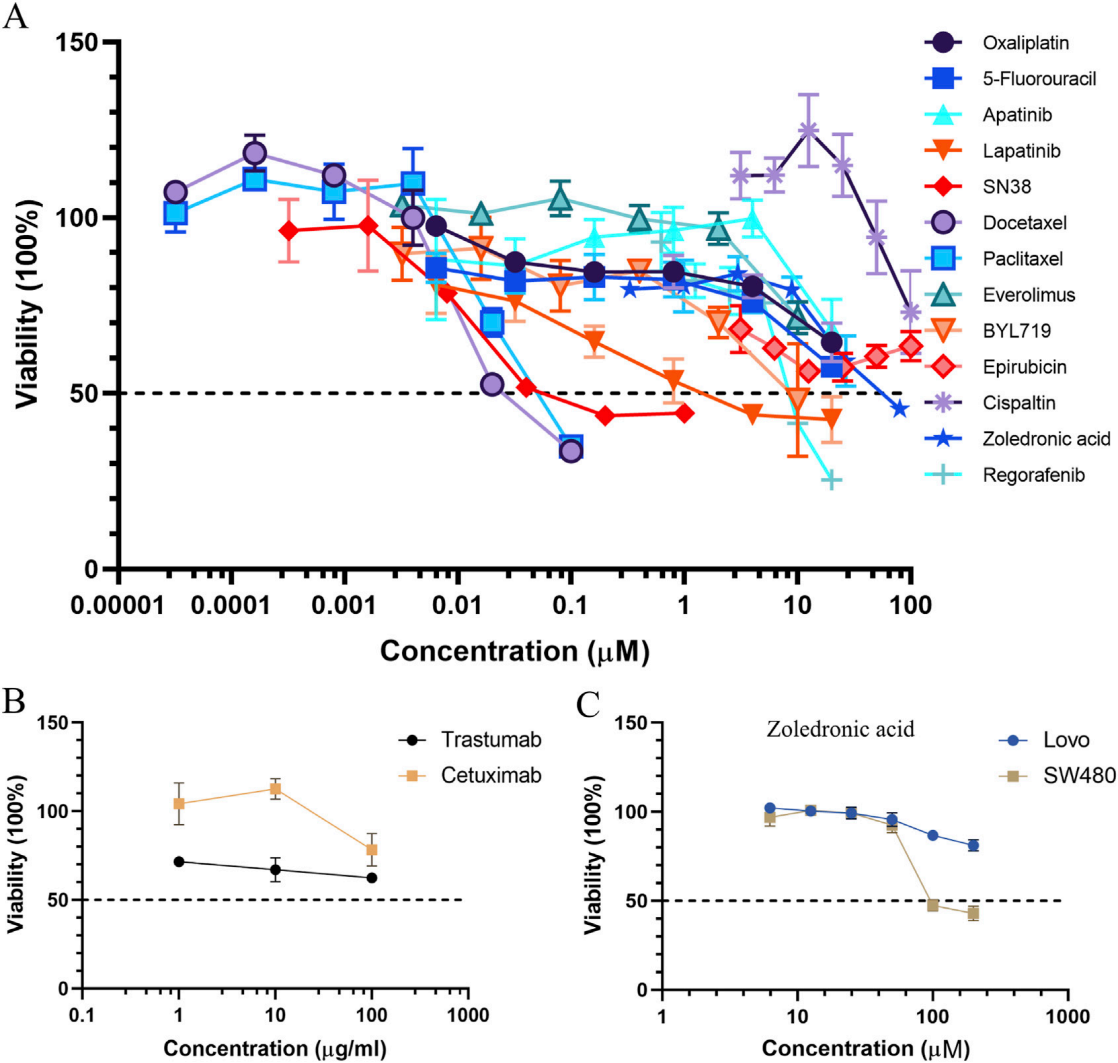
Curves of the organoid treated with chemotherapeutic drugs, targeted medicines **(A)**, and monoclonal antibodies **(B)**. Dose–response curves of colon cancer cell lines to zoledronic acid **(C)**.

Among these drugs, the IC_50_ values of each drug were calculated from the respective dose–response curves, which were 29.35 μM for oxaliplatin, 20.61 μM for 5-fluorouracil, 0.1045 μM for SN38, 35.83 nM for docetaxel, 57.45 nM for paclitaxel, 1.58 μM for lapatinib, 6.67 μM for BYL719, 8.78 μM for regorafenib, 26.98 μM for everolimus, and 47.32 μM for apatinib. However, the predicted IC_50_ values for trastuzumab and cetuximab were over 100 μg/mL. The IC_50_ values of each compound are summarized in Table 1. Dose–response curves are displayed in Figure 9A. As shown in Figure 9A, the sigmoid colon cancer organoid demonstrated multiple drug resistance, such as to oxaliplatin, 5-fluorouracil, and cisplatin. In addition, the sigmoid colon cancer organoid was insensitive to monoclonal antibody therapies (Figure 9B). To identify the antitumor activity of zoledronic acid, the sigmoid colon cancer organoid, SW480, and LoVo cell lines were used for drug sensitivity tests (Figure 9C). The IC_50_ values of the organoid, SW480, and LoVo were 58.96 μM, 171.5 μM, and over 200 μM, respectively (Table 2).

**TABLE 1:** IC50 values for the sigmoid colon cancer organoid.

Drug	IC_50_ (μM)	95% confidence interval
Oxaliplatin	29.35	17.66 to 52.87
5-Fluorouracil	20.61	10.83 to 41.97
SN38	0.1045	0.03647 to 0.3269
Docetaxel	0.03583	0.02170 to 0.06068
Paclitaxel	0.05745	0.03870 to 0.08671
Cisplatin	>100	NA
Epirubicin	>100	NA
Lapatinib	1.578	0.3565 to 7.219
BYL719	6.674	3.691 to 12.32
Regorafenib	8.781	7.178 to 10.78
Everolimus	26.98	20.93 to 36.11
Apatinib	47.32	26.99 to 102.0
Zoledronic acid	58.96	44.06 to 80.26
Trastuzumab	>100 μg	NA
Cetuximab	>100 μg	NA

**TABLE 2:** IC_50_ values of zoledronic acid for the colon cancer cell lines.

Drug	IC_50_ (μM)	95% confidence interval
SW480	171.5	125.5 to 241.3
LoVo	>200	NA

In conclusion, docetaxel was found to be the most sensitive drug for the sigmoid colon cancer organoid.

## Discussion

Although the spine is a common location of metastasis due to malignant tumors, CRC patients with bone metastasis are very rare. The original sites of bone metastasis are largely derived from prostate, breast, and lung cancer (Kawamura et al., 2018). CRC patients with bone metastases have poor quality of life and prognosis. It has been reported that the 5-year overall survival rate of CRC patients with bone metastases is lower than 5% (Kawamura et al., 2018). Effective drug treatment for this type of terminal-stage patient is limited. However, tumor organoids with a three-dimensional structure derived from cancer stem cells are regarded as the best model to predict drug response as they maintain the heterogeneity of the original tumor (Tuveson and Clevers, 2019). Here, we successfully established an organoid line of sigmoid colon cancer with bone metastasis. Then, we evaluated the recapitulation of the histopathology of the primary tumor tissue.

In addition, we isolated genomic DNA for sequencing. For both somatic mutation and germline mutation analysis, we found *AHNAK2*, *FLG*, *MUC19*, and *MUC16* among the top five mutation genes (Figures 5F, 6F). High-frequency mutation of *AHNAK2* correlated with poor prognosis of cancer patients, promoting epithelial–mesenchymal transition and activating oncogenic pathways, such as MAPK, Wnt, and MEK signaling pathways (Zhang et al., 2023). It was also reported that AHNAK2 mutation remodeled the tumor microenvironment, including elevating the infiltration of M1 macrophages, B cells, and fibroblasts, leading to impaired response to chemotherapy (Ye et al., 2024). In colon cancer, FLG mutation was associated with an increased risk of death due to translocation of gut bacteria and dysregulation of the immune response (Ge et al., 2020). The coding proteins of the MUC family members MUC16 and MUC19 were two different types of proteins. MUC16 (CA125) was a cross-membrane protein. MUC16 was regarded to be an effective biomarker in the detection of pancreatic cancer, gastric cancer, and colorectal cancer (Zhang et al., 2016). MUC19 was a secreted protein. However, there are limited studies regarding the relationship between MUC19 mutation and colorectal cancer. Chu’s study also reported that MUC19 mutations can be detected in the case of colorectal cancer (Chu et al., 2019), similar to the sigmoid colon cancer patient of our study. Driver gene identification was significant for targeted therapy. Our present study identified SLC9B1, PDZRN3, PABPC3, ASPM, and ZNF419 alterations in our patient (Supplementary Figure S1). Among these genes, *ASPM* was confirmed to promote colon cancer progression (Yang et al., 2021). Targeting these genes may be beneficial for the patient.

Next, the sigmoid colon cancer organoid was used for drug screening to identify the potentially effective drugs. In this case, the patient was a young woman diagnosed with synchronous bone metastasis with sigmoid colon cancer. The incidence rate of bone metastasis of the left colon cancer was reported to be higher than that of the right colon cancer (Baek et al., 2016; Zhenghong et al., 2017; Kawamura et al., 2018). After MDT discussion, the patient started to accept chemotherapy (including FOLFOX combined with the bevacizumab regimen and XELOX combined with the bevacizumab regimen). However, according to the response evaluation criteria in solid tumors (RECIST) principle, the chemotherapy response of the tumor showed no significant change (Litiere et al., 2017). In order to avoid complications during subsequent chemotherapy, the patient underwent palliative surgery to remove the primary tumor. Unfortunately, in the postoperative recovery period, wound infection and bone pain occurred, which delayed the start of chemotherapy. Thus, on 21 April 2020, the patient accepted postoperative chemotherapy (FOFIRI combined with bevacizumab). Although CRC patients with bone metastases face difficulties in both treatment and poor prognosis, chemotherapy regimens such as XELOX, FOLFOX, and FOLFIRI can help improve survival (Naito et al., 2014). However, it is difficult to identify an effective chemotherapy regimen in terminal-stage patients with limited survival. A tumor organoid, a 3D culture of cancer cells, has been regarded as an excellent drug screening platform for precision medicine (Walsh et al., 2017; Weeber et al., 2017). Many studies have reported that cancer organoids can recapitulate the heterogeneity of the original tumor (Lee et al., 2018; Sachs et al., 2018; Yan et al., 2018; Boretto et al., 2019). Among the current disease models, cancer cell lines are utilized widely with the lowest cost. Meanwhile, it is difficult for cell lines to maintain tumor heterogeneity. Thus, cancer cell lines may not be suitable for establishing a drug screening model. The patient-derived xenograft (PDX) model, a relatively higher quality cancer model than cancer cell lines, has shown superiority as a preclinical model in drug screening and clinical trials (Jung et al., 2018). However, ethical issues and high costs have limited the development of the PDX model. The majority of cancer organoids are derived from surgical or biopsy specimens, which do not cause secondary damage to the patients. In addition, cancer organoids can be used for expanding and high-throughput research, which is even more convenient and inexpensive than conducting preclinical drug testing using the PDX model, (Tuveson and Clevers, 2019). In summary, the cancer-derived organoid is the best pre-clinical model for drug screening.

Here, we obtained tumor tissue and cultured the sigmoid colon cancer organoid on the day of surgery. Then, we successfully established the sigmoid colon cancer organoid line, which could expand for a long period. The postoperative pathology report confirmed that the tumor was MSI-H. From our IHC and genomic sequencing results, the sigmoid colon cancer organoid captures the histopathological characteristics and molecular mutation patterns of the original sigmoid colon cancer cells well (Figures 3–7).

SN38, a topoisomerase I inhibitor and the active form of irinotecan, exhibits a better anticancer effect than 5-FU and oxaliplatin on the sigmoid colon cancer organoid. From our drug screening test and response to chemotherapy of the patient, we can draw a conclusion that the original tumor was not sensitive to first-line chemotherapy drugs and the patient did not benefit from preoperative chemotherapy. In addition, we deduce that the FOLFIRI regimen may help reduce the tumor burden of the patient.

Zoledronic acid, a new-generation nitrogen-containing bisphosphonate, is usually used to treat osteoporosis and osteolytic bone metastasis to prevent skeletal-related events. It is an osteoclast inhibitor, inhibiting the activity of farnesyl diphosphate synthase, which is the key enzyme of the mevalonate pathway. Recently, many research studies have reported that zoledronic acid has direct and indirect anticancer activities (Gnant and Clezardin, 2012; Finianos and Aragon-Ching, 2019; Liu et al., 2019). In *in vivo* and *in vitro* models, in addition to its anti-osteoclastic activity, zoledronic acid has been proven to affect cancer cell proliferation, invasion, migration, and angiogenesis. Here, the patient was a case with bone metastasis. From the treatment line (Figure 1A), zoledronic acid was used in preoperative chemotherapy. We tried to examine the antitumor effect on the sigmoid colon cancer organoid and colon cancer cell lines. According to the result, the response of zoledronic acid (IC_50_: 58.96 μM) was more sensitive in the sigmoid colon cancer organoid than in LoVo and SW480 cell lines (Figure 9C). Unfortunately, due to the advanced stage of the disease and multiple spinal metastases, specimens of spinal metastases were of little significance for biopsy. To some extent, zoledronic acid may be a better choice for patients with early-stage or single-site bone metastases.

Docetaxel and paclitaxel are the first generation of taxane anticancer agents. They work by binding to tubulins/microtubules, which play an important role in cell division (Ojima et al., 2016). FDA-approved docetaxel and paclitaxel are used for various kinds of solid tumors, including breast cancer, non-small cell lung cancer, prostate cancer, and head and neck cancer (de Weger et al., 2014). In our drug screening test (Table 1), the response of docetaxel (IC_50_, 0.03583 μM) and paclitaxel (IC_50_, 0.05745 μM) to the sigmoid colon cancer organoid seemed to be more sensitive than other kinds of chemotherapeutic drugs. Thus, compared to the response of the first-line antitumor drugs, such as 5-FU (IC_50_, 20.61 μM) and oxaliplatin (IC_50_, 29.35 μM), our results show that docetaxel and paclitaxel may be alternative choices for our sigmoid colon cancer patient.

Among other types of molecularly targeted small-molecule anticancer drugs we used, lapatinib (IC_50_, 1.578 μM) was the most sensitive treatment for our patient (Table 1). The response of monoclonal antibodies (IC_50_ of trastuzumab and cetuximab over 100 μg) (Figure 9B) as a single drug treatment for the sigmoid colon cancer organoid is not significant.

There are some limitations to our study. First, we did not obtain the spinal metastases specimen of the patient. In general, primary tissues of colorectal cancer and metastasis of colorectal cancer exhibit high genomic consistency, including KRAS, NRAS, and BRAF mutations, and driver genes of APC, TP53, and PIK3CA (Brannon et al., 2014). For MSI status, colorectal cancer primary tumors and metastases were highly matched (Deschoolmeester et al., 2010). However, it has been reported that approximately 40% of primary and metastatic tumors have different somatic mutation profiles (Grellety et al., 2017). This may be explained by the tumor heterogeneity of the initial surgical resection specimen being different from the later biopsy from the metastatic site. Another reason was the tumor microenvironment heterogeneity between the primary and metastatic tumor, such as different immune cell infiltration, cancer-associated fibroblasts, and extracellular matrix (Khaliq et al., 2024). It may lead to distinct treatment outcomes between the primary site and the metastatic site. For precision medicine, an *in vitro* assay drug screening test of the patient-derived organoid from the metastatic site may provide potential benefits for cancer patients (Jensen et al., 2023). Thus, our drug screening test may not directly reflect the response to spinal metastases. Second, we conducted drug screening on just one sigmoid colon cancer organoid. Our result only revealed the characteristics of this specific case with this particular patient. More CRC patients with bone metastases should be incorporated into the study. Third, although the sigmoid colon cancer organoid served as a stable *in vitro* drug screening model, the efficacy of the preclinical response still needs to be evaluated.

## Conclusion

To conclude, we have successfully established an *in vitro* model of sigmoid colon cancer with spinal metastases and demonstrated a favorable concordance between the cancer organoid and original tumor cells in histopathology and genomic landscape. The sigmoid colon cancer organoid also provided a preclinical model for drug screening. We found several drugs, such as docetaxel, paclitaxel, and lapatinib, which were regarded as potentially effective drugs for personalizing the treatment of sigmoid colon cancer with bone metastasis.

## Data Availability

The original contributions presented in the study are included in the article/Supplementary Material; further inquiries can be directed to the corresponding authors.

## Supplementary material

SUPPLEMENTARY FIGURE S1 Driver gene analysis of blood, tissue, and organoid samples (left to right).

SUPPLEMENTARY TABLE S1 Organoid culture medium formulation.
