# Supplementary material for: Establishment and characterization of a sigmoid colon cancer organoid with spinal metastasis
Source: Front Cell Dev Biol. 2025 Jan 3;12:1510264. doi: 10.3389/fcell.2024.1510264 (PMC11739105; doi:10.3389/fcell.2024.1510264)
Supplement: Supplementary file 1 [file Table1.docx]

Table S1. Organoid culture medium

| Component | Source (catalog number) | Concentration |
| --- | --- | --- |
| Advanced DMEN/F12 | Gibco (12634010) | 1x |
| HEPES buffer | Biosharp (BL1061A) | 10mM |
| Penicillin-Streptomycin | Gibco (15140122) | 50U/ml |
| GlutaMAX | ThermoFisher (35050061) | 1x |
| Noggin | NovoProtein (C028) | 100 ng/ml |
| N-acetylcysteine (NAC) | MCE (HY-B0215) | 1mM |
| B-27 | Gibco (17504-044) | 1x |
| A83-01 | MCE (HY-10432) | 500nM |
| SB 202190 | MCE (HY-10295) | 10µM |
