# Supplementary figures and images for: Establishment and characterization of a sigmoid colon cancer organoid with spinal metastasis
Source: Front Cell Dev Biol. 2025 Jan 3;12:1510264. doi: 10.3389/fcell.2024.1510264 (PMC11739105; doi:10.3389/fcell.2024.1510264)

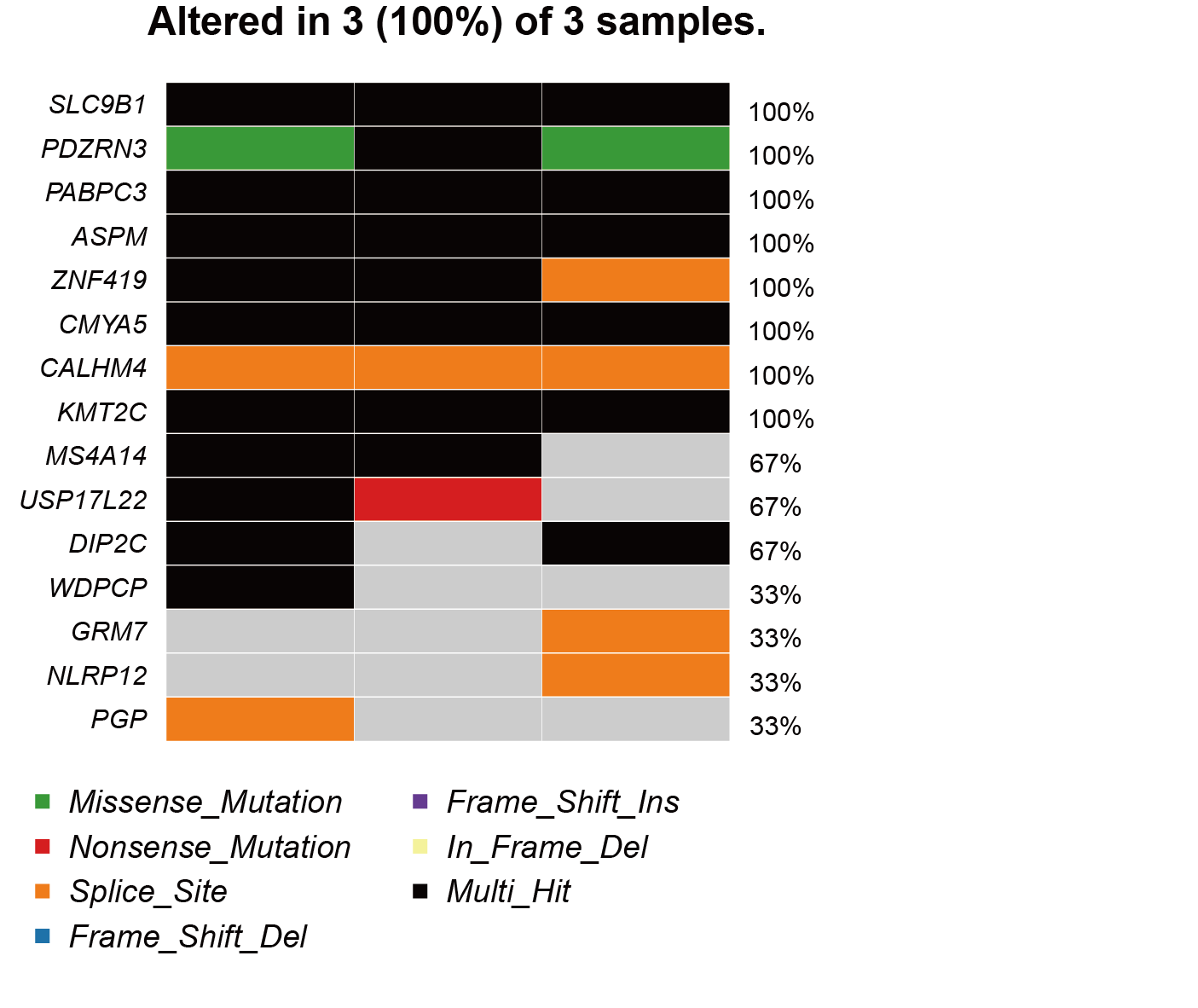

Supplement: Supplementary file 2 [file Image1.tif]
